# Association between history of pulmonary tuberculosis and chronic obstructive pulmonary disease: a systematic review and meta-analysis

**DOI:** 10.3389/fmed.2026.1926128

**Published:** 2026-08-10

**Authors:** Reyhangul Aken, Yanchun Li, Xia Huang, Nadire Abudusalamu, Yan Chen, Zhiping Chen, Yuanyuan Li

**Affiliations:** 1 Department of Tuberculosis, The Sixth People's Hospital of Xinjiang Uygur Autonomous Region, Ürümqi, China; 2 Department of Respiratory and Critical Care Medicine, The Sixth People's Hospital of Xinjiang Uygur Autonomous Region, Ürümqi, China; 3 The Eighth Clinical Medical College, Xinjiang Medical University, Ürümqi, China

**Keywords:** COPD, meta-analysis, pulmonary TB, systematic review, tuberculosis

## Abstract

**Background:**

Survivors of pulmonary tuberculosis (TB) frequently report chronic respiratory symptoms; however, the magnitude of the association with chronic obstructive pulmonary disease (COPD) and the reciprocal burden of COPD and TB in each population remain unclear. Hence, this review was conducted to determine the association between a history of pulmonary tuberculosis and chronic obstructive pulmonary disease.

**Methods:**

Following PRISMA 2020, we searched MEDLINE, Embase, Web of Science, and Scopus from inception to August 2025 for analytical observational studies comparing individuals with and without a history of pulmonary TB. Odds ratios (ORs) were pooled using random-effects inverse-variance models (DerSimonian–Laird). Heterogeneity (*I*^2^ and *τ*^2^) and small-study effects (using Egger’s test) were assessed. Prevalence meta-analyses were conducted to estimate the following: (i) COPD among pulmonary TB survivors and (ii) the prevalence of prior pulmonary TB among people with COPD.

**Results:**

Forty studies met the inclusion criteria. Twenty-six studies (*n* = 1,044,562) contributed to the association analysis: prior pulmonary TB was associated with higher odds of COPD (pooled OR = 2.65, 95% CI: 2.14–3.28), with substantial heterogeneity (*I*^2^ = 94.9%). When stratified by study design, pooled ORs were 4.92 for case–control studies, 2.64 for cross-sectional studies, and 2.46 for cohort studies. Across regions, positive associations were found in Asia (2.21), Africa (9.36), the Americas (3.60), and Europe (2.77). Egger’s test indicated small-study effects (*p* = 0.013), but sensitivity analyses showed that the pooled estimates were reliable. The pooled prevalence of COPD among survivors of pulmonary TB was 34% (95%CI: 28–40%). Among COPD patients, 17% (95% CI: 10–24%) reported a history of pulmonary TB.

**Conclusion:**

A history of pulmonary TB was found to be a strong and consistent risk factor for COPD across different designs and regions, with a significant bidirectional burden. Integrating post-TB lung assessment (including spirometry), targeted COPD management, and risk-reduction strategies into TB programs is required.

**Systematic review registration:**

https://www.crd.york.ac.uk/PROSPERO/view/CRD420261415397, PROSPERO registration number is CRD420261415397.

## Introduction

Chronic obstructive pulmonary disease (COPD) remains a leading cause of morbidity and mortality worldwide, with 392 million adults affected in 2019. Disproportionate burdens are seen in low and middle-income countries. While tobacco smoke and biomass exposure are considered major risk factors, there has been growing recognition of non-smoking causes that vary by geography and across the life course. Amongst these, prior pulmonary tuberculosis (TB) has emerged as a clinically important, preventable determinant of chronic airflow limitation, especially in settings where TB is endemic and the prevalence of smoking is changing. This epidemiological overlap raises critical questions about the contribution of TB survivors to the burden of COPD and the disease phenotypes found in routine care (1–3). Population surveys have demonstrated a high prevalence of small-airway dysfunction and spirometric obstruction among adults with a history of pulmonary TB, including never smokers, underscoring that pulmonary TB can lead to COPD through pathways that are distinct from smoking-related emphysema (4, 5).

Observational studies over the past two decades have indicated a significant association between prior pulmonary TB and COPD. Comprehensive meta-analysis reported substantially higher odds of COPD among individuals with a TB history compared with those without across diverse designs and regions. More recently, high-quality cohort studies have strengthened causal inference by documenting elevated *incident* COPD risk after PTB. In a nationwide Korean cohort of TB survivors, the risk of developing COPD during follow-up was significantly higher than that among matched comparators after adjustment for smoking and other confounders. Consistent with these findings, large UK Biobank analyses have found that self-reported prior pulmonary TB is associated with a twofold increase in incident COPD over a median of 12 + years, with consistent association across various variables, such as age, sex, adiposity, smoking status, and polygenic risk strata. Together, these data suggest that pulmonary TB is not merely a correlate of low lung function but may be an independent cause of COPD (6–8).

Clinically, TB-associated COPD may present with features that differ from usual smoking-related disease conditions. Imaging studies and physiological studies suggest that a phenotype marked by bronchiectasis and small-airway involvement, variable distribution of emphysema, and, in a few reports, lower bronchodilator response have implications for therapeutic and prognostic pathways. Importantly, multi-country population-based studies from TB endemic settings have shown that a previous history of pulmonary TB is an under-recognized risk factor for airflow obstruction and poorer lung function, emphasizing the need for post-TB respiratory surveillance and tailored COPD management algorithms in high-burden regions. Finding prior history of pulmonary TB as a specific exposure in risk stratification of COPD could refine prevention, case-finding, and long-term care for millions of survivors of TB worldwide (5, 9). Hence, this review was done to determine the association between history of pulmonary tuberculosis and chronic obstructive pulmonary disease.

## Methods

### Eligibility criteria

We used PECOS criteria as follows:

*Population*: Humans of any age from community or clinical settings.

*Exposure*: History of pulmonary tuberculosis (PTB)—ascertained by medical record/registries or clinician diagnosis, microbiologic confirmation, characteristic radiographic/CT sequelae consistent with healed PTB, or self-report. We prioritized sources in the order listed for risk-of-bias assessment. Studies exclusively addressing extrapulmonary TB without pulmonary involvement were excluded.

*Comparator*: Individuals without any history of PTB (explicitly stated or reasonably inferred from cohort/registry definitions).

*Outcome*: Chronic obstructive pulmonary disease (COPD) defined by (i) post-bronchodilator FEV₁/FVC < 0.70 or below lower limit of normal, (ii) physician diagnosis/ICD codes, or (iii) validated self-report.

*Study designs*: Observational cohort (prospective/retrospective), case–control, and analytical cross-sectional studies. We excluded case reports/series, narrative reviews, editorials, mechanistic-only studies, and studies without a non-PTB comparator.

### Information sources

We searched MEDLINE (via PubMed), Embase (via Ovid), Web of Science Core Collection, and Scopus from inception to 25 August 2025. To reduce reporting bias, we also searched WHO Global Index Medicus and screened reference lists of included studies and relevant reviews. We explored clinical trial registries (ClinicalTrials.gov, WHO ICTRP) for observational cohorts with registry records and contacted authors for unpublished effect estimates when necessary.

### Search strategy

A librarian-reviewed strategy combined controlled vocabulary and text words for tuberculosis and COPD with validated spirometry terms. No design or language filters were applied. Database-specific strategies (Embase Emtree terms, Scopus/WoS field tags) were adapted from this scaffold, provided in the Supplement. We exported all records to a reference manager and removed duplicates prior to screening.

### Selection process

Two reviewers independently screened titles/abstracts in Rayyan after pilot calibration (100 records; Cohen’s *κ* targeted ≥0.80). Potentially eligible records underwent full-text review in duplicate. Disagreements were resolved by consensus or a third reviewer. We recorded exclusion reasons at full-text and presented the study flow in a PRISMA 2020 flow diagram.

### Data collection process

A piloted, standardized extraction form (Excel) captured study and effect-estimate details. Two reviewers extracted data independently with adjudication by a senior reviewer. For missing or unclear information (e.g., COPD definition and adjustment covariates), we contacted corresponding authors (two attempts over 3–4 weeks).

### Data items

*Study characteristics*: Country, WHO region/World Bank income level, setting, study period, design, sampling frame, sample size, and follow-up (if cohort).

*Population*: Mean/median age, sex distribution, smoking status (never/ever/current), biomass/occupational exposures where available.

*Exposure (PTB)*: Ascertainment method (microbiologic/clinical/radiographic/self-report), time since TB treatment completion, drug resistance, and extent of disease (if reported).

*Outcomes*: COPD definition/ascertainment.

*Effect measures and adjustments*: OR, RR, HR with 95% CIs; covariates in adjusted models (minimum set: age, sex, smoking). Where necessary, we derived effect sizes from raw 2 × 2 data.

### Study risk of bias assessment

We assessed non-randomized studies using ROBINS-E (bias due to confounding, selection of participants, classification of exposure, deviations from intended exposures, missing data, outcome measurement, selection of reported results) (10). For purely cross-sectional analyses without a temporal component, we supplemented with the JBI checklist for analytical cross-sectional studies (11). Two reviewers rated risk of bias independently after calibration; disagreements were resolved by consensus.

### Statistical analysis

For dichotomous COPD outcomes, the principal measure was odds ratio (OR). Effect sizes were log-transformed with corresponding standard errors. We retrieved the raw counts of events and participants in each group to obtain the OR with 95% confidence interval (CI). We anticipated between-study heterogeneity and prespecified random-effects meta-analysis using restricted maximum likelihood (REML) with Hartung–Knapp adjustments for confidence intervals. We reported the τ^2^ (between-study variance), I^2^, and Cochran’s Q (12). Subgroup analyses were performed based on study design (cohort vs. case–control vs. cross-sectional) and WHO region. Leave-one-out sensitivity analyses were done to determine the robustness of the estimates. Primary analyses will be conducted in Stata 17 software. For syntheses with ≥10 studies, we have inspected funnel plots and tested for small-study effects using Egger’s regression.

## Results

**Figure 1:**
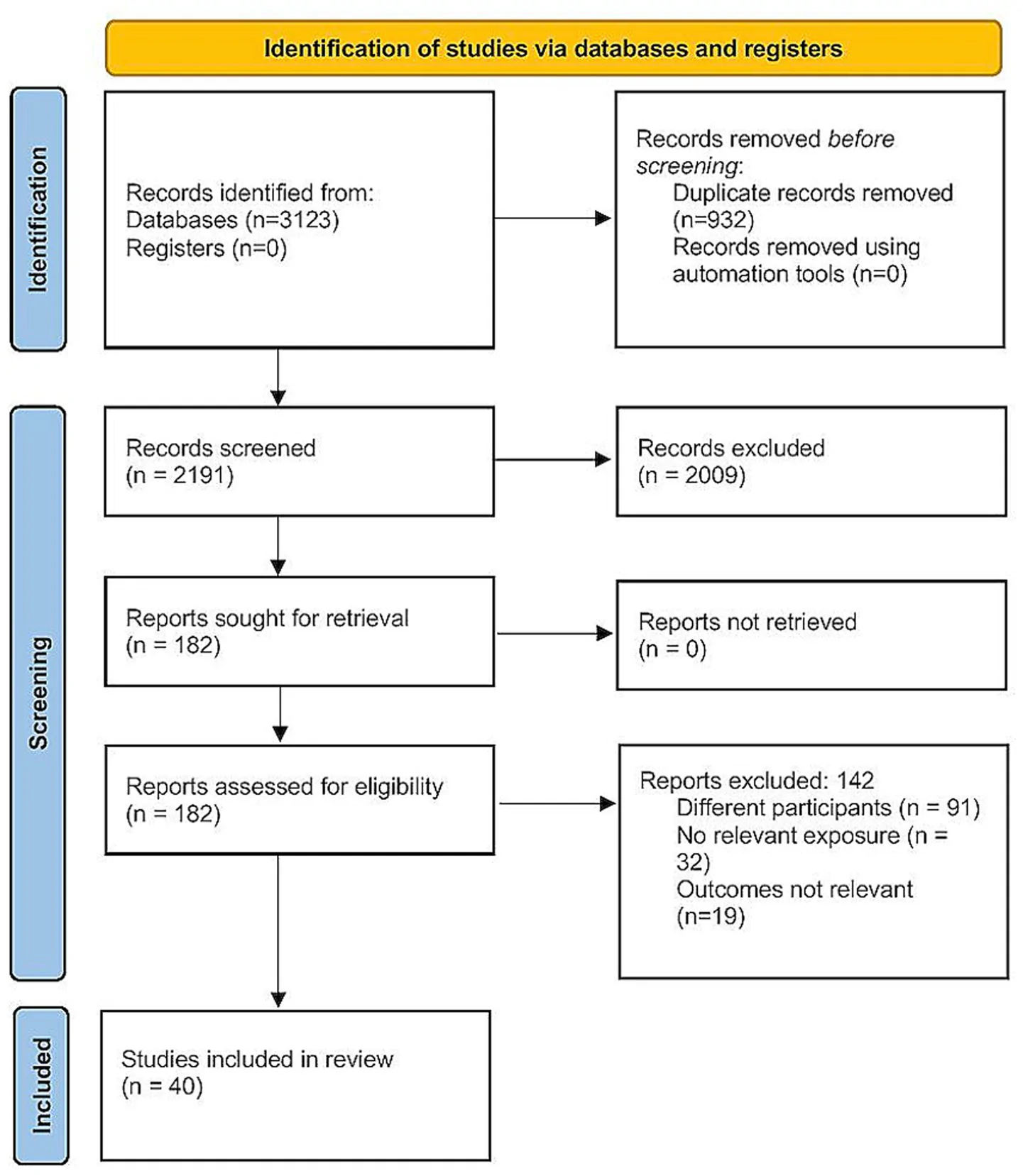
PRISMA flowchart.

From 3,123 database records, 932 duplicates were removed, leaving 2,191 records for screening; 2,009 were excluded. We assessed 182 full texts (none unretrieved) and excluded 142, that is, different participants (*n* = 91), no relevant exposure (*n* = 32), and outcomes not relevant (*n* = 19). Approximately 40 studies were included in the review (Figure 1) (7–9, 13–49).

### Characteristics of the included studies

The review synthesized 40 studies spanning Asia, Africa, Europe, and the Americas, predominantly cross-sectional designs (28/40; 70%), with 10 cohort studies (25%) and 2 case–control studies (5%). Sampling frames ranged from community surveys and non-institutionalized adult cohorts to clinic-based COPD populations and post-tuberculosis (TB) follow-up cohorts. Sample sizes varied markedly from 25 participants in a clinic series to >300,000 in a population cohort providing both breadth and depth across settings. Most studies defined COPD using spirometry (FEV₁/FVC < 70%), sometimes alongside GOLD criteria or LLN thresholds; a minority relied on ICD-coded diagnoses, physician diagnosis, chronic bronchitis criteria, or symptom tools (e.g., CAT). TB exposure/diagnosis was predominantly self-reported, with several studies incorporating chest X-ray, computed tomography, medical records, or microbiological confirmation. Where reported, mean ages clustered from the mid-40s to late-70s, with TB-associated COPD groups generally comparable to or slightly older than other COPD groups. Risk of bias assessments ranged from low (14 studies—often large, population-based cohorts with standardised spirometry and administrative case definitions), moderate (13 studies) to high (13 studies—typically small, single-centre cross-sectional studies), reflecting heterogeneity in design quality, outcome ascertainment, and confounder control (Table 1).

**Table 1:** Characteristics of included studies (*N* = 40).

Author	Country	Study design	Population	Sample size	COPD definition	TB definition	Overall mean age	TB associated COPD patients mean age	Other COPD patients mean age (in years)	Risk of bias
Aggarwal et al. (13)	India	Case–control	Patients with stable COPD who attended a chest outpatient department, with similar age and gender distribution, were recruited as controls	148	FEV1/FVC < 70%	TB history (self-reported)	60.2 ± 8.9	60.2 ± 8.9	61.8 ± 8.5	Moderate
Choi et al. (18)	Korea	Cross-sectional	Participants aged 40 years or older who underwent chest X-ray (CXR) and spirometry	13,522	FEV1/FVC < 70%	Self-reported history of physician-diagnosed TB or TB lesions on CXR	NA	NA	NA	Moderate
Dutta and Deshmukh (20)	India	Cross-sectional	Women aged 40 years or more	1,650	Chronic Bronchitis	TB history (self-reported)	NA	NA	NA	High
Echazarreta et al. (21)	Argentina	Cross-sectional	Men and women aged 40 years or older	3,469	FEV1/FVC < 70%	TB history (self-reported)	58.8 ± 11.6	NA	NA	High
Fang et al. (22)	China	Cross-sectional	Individuals aged 40 years or older	66,752	FEV1/FVC < 70%	TB history (self-reported)	54.9 ± 11.1	NA	NA	Low
Gemert et al. (23)	Uganda	Cross-sectional	People above the age of 30 years from 30 villages between	609	FEV1/FVC < 70%	TB history (self-reported)	45.2 ± 13.6	46.7 ± 14.0	NA	Moderate
Govender et al. (24)	South Africa	Case–control	COPD patients diagnosed by physician were included	212	Diagnosed by pulmonologist	TB history (self-reported)	NA	NA	NA	Moderate
Guiedem et al. (25)	Cameroon	Cohort study	Men and women aged 25 to 80 were included	92	FEV1/FVC < 70%	TB history (self-reported)	NA	43 ± 12.38	NA	Low
Guiedem et al. (26)	Cameroon	Cohort study	Patients with COPD and matched controls were included	150	Chronic Bronchitis, FEV1/FVC < 70%	TB history (self-reported)	NA	40 ± 2.1	43 ± 12.38	Low
Hagstad et al. (27)	Sweden	Cross-sectional	Adults aged 20–77 years were involved in the study	1897	Bronchodilator ratio of FEV1/FVC < 70%	TB history (self-reported)	NA	NA	NA	High
Hulya et al. (28)	Turkey	Cross-sectional	Patients who attended the chest diseases outpatient clinic	172	The COPD assessment test was used to capture COPD patients	TB history (self-reported)	NA	NA	NA	High
Idolor et al. (29)	Philippines	Cross-sectional	Non-hospitalised men and women, aged 40 years or older	722	FEV1/FVC < 70%	TB history (self-reported)	NA	62.02 ± 11.05	52.14 ± 9.46	Moderate
Jin et al. (31)	China	Cross-sectional	Patients with stable COPD	231	COPD was based on the Global Initiative for Chronic Obstructive Lung Disease (GOLD) criteria	PTB was defined based on computed tomography (CT) scan findings	76.8 ± 9.4	76.8 ± 9.4	NA	High
Lamprecht et al. (33)	China, Turkey, Austria, South Africa, Iceland, Germany, Poland, Norway, Canada, United States, Philippines, Australia, London, and Sweden.	Cohort	Non-institutionalised adults aged ≥ 40 years	10,000	FEV1/FVC < 70%, lower limit of normal (LLN)	TB history (self-reported)	NA	NA	NA	Low
Lee et al. (34)	Korea	Cross-sectional	Civilian, non-institutionalised Korean adults aged 18 years and older	3,687	FEV1/FVC < 70%, lower limit of normal (LLN)	TB history by CXR	43.4	NA	42.5 ± 14.0	Low
Magitta et al. (35)	Tanzania	Cross-sectional	Adults aged ≥35 years	496	FEV1/FVC < 70%	TB history (self-reported)	NA	NA	NA	Moderate
Mahmood et al. (36)	India	Cross-sectional	Chronic obstructive pulmonary disease (COPD) patients, aged >18 years of either gender	200	Global initiative for chronic obstructive lung disease (GOLD) guidelines	Medical History	NA	NA	NA	Moderate
Nishida et al. (37)	Japan	Cross-sectional	Japanese patients aged 40 years or older who had undergone spirometry tests	11,898	FEV1/FVC < 70%	TB history (self-reported)	NA	69.06 ± 10.53	55.34 ± 17.64	Low
Nugmanova et al. (38)	Ukraine, Kazakhstan and Azerbaijan	Cross-Sectional	Adult subjects who were ≥18 years old	2,842	Global Initiative for Chronic Obstructive Lung Disease (GOLD) guidelines, FEV1/FVC < 70%	TB history (self-reported)	NA	NA	NA	Moderate
Park et al. (40)	Korea	Cohort study	Adults aged ≥40 years old who had been diagnosed with COPD	1784	FEV1/FVC < 70%	TB history (self-reported) and CXR	NA	73.2 ± 27.9	NA	Low
Shettar et al. (42)	India	Cross-sectional	Previously treated pulmonary tuberculosis (PTB) patients and patients with COPD	116	previously treated pulmonary tuberculosis (PTB) patients and patients with COPD	TB history (self-reported)	NA	NA	NA	High
Smith et al. (43)	China	Cross-sectional	Male and female participants aged 30–79 years	317,399	FEV1/FVC < 70%	TB history (self-reported)	54.4 ± 11.9	NA	NA	High
Nguyen Viet et al. (44)	Vietnam	Cross-sectional	Participants were female or male) non-smokers, 40 years or older and able to perform a spirometry test.	1,506	Global initiative for chronic obstructive lung disease (GOLD) guidelines	TB history (self-reported)	53.9 ± 0.3	59.0 ± 1.6	NA	High
Wang et al. (45)	China	Cross-sectional	Adults aged 20 years or older from ten provinces	50,991	Global initiative for chronic obstructive lung disease (GOLD) guidelines, FEV1/FVC < 70%	TB history (self-reported)	43.6	NA	NA	Low
Zeng et al. (8)	London	Cohort	Adults aged 40–69 years	216,130	FEV1/FVC < 70%	TB history (self-reported)	NA	NA	NA	Moderate
Caballero et al. (16)	Colombia	Cross-sectional	Civilian, noninstitutionalised adults of both genders aged 40 years or older	5,539	FEV1/FVC < 70%	TB history (self-reported)	55.8 ± 11.2	NA	NA	High
Yakar et al. (47)	Turkey	Cross-sectional	Patients hospitalised with COPD exacerbation	598	FEV1/FVC < 70%	TB history (self-reported)	69.5 ± 10.6	66.3 ± 11.3	NA	Low
Park et al. (41)	Korea	Cohort study	Men and women aged between 50 and 84 years diagnosed with COPD	47,632	Presence of the International Classification of Diseases, Tenth Revision (ICD-10) code J43 or J44	TB history—CXR	NA	66.3 ± 8.4	65.3 ± 8.3	Low
Inghammar et al. (30)	Sweden	Cohort study	40 years of age or older who had been discharged with a diagnosis of COPD	115,867	International Classification of Diseases, Ninth and Tenth revisions (ICD-9: 491–492, 496; ICD-10: J41–J44)	TB history (self-reported)	NA	NA	NA	Low
Osman (39)	Sudan	Cross-sectional	Past Pulmonary Tuberculosis (PPTB) subjects and their age and sex-matched controls	272	FEV1/FVC < 70%	TB history (self-reported)	42.22 ± 8.55	43.97 ± 8.51	44.47 ± 8.61	High
Zamzam et al. (49)	Egypt	Cross sectional	40 years of age or older who had been discharged with a diagnosis of COPD	231,734	International Classification of Diseases (ICD), Ninth and Tenth revisions (ICD-9: 491–492, 496; ICD-10: J41–J44)	TB history (self-reported)	NA	75.2	77.8	High
Balan and Kumar (14)	India	Cross-sectional	All diagnosed Pulmonary Tuberculosis (TB) registered patients	178	FEV1/FVC < 70%	Microbiologically confirmed or clinically diagnosed	NA	NA	NA	Moderate
Bekele et al. (15)	Ethiopia	Cross-sectional	Consecutive Chest Unit patients aged > 15 years who had previously been successfully treated for PTB	99	FEV1/FVC < 70%	TB history (self-reported)	42.7	NA	NA	Moderate
Das et al. (19)	India	Cross-sectional	Consecutive subjects who had pulmonary tuberculosis	100	FEV1/FVC < 70%	TB history (self-reported)	53.27	NA	NA	Moderate
Joo et al. (32)	Korea	Cohort study	Patients who had been treated with anti-tuberculosis medication for over 6 months and had both pre-TB and post-TB spirometry were enrolled	750	FEV1/FVC < 70%	TB history (self-reported)	NA	NA	NA	Low
Kamenar et al. (9)	United States	Cross-sectional	Participants with previous tuberculosis disease	12,396	FEV1/FVC < 70%	TB history (self-reported)	NA	NA	NA	High
Kim et al. (7)	Korea	Cohort study	Patients survived TB were included with their age and sex matched control	62,066	FEV1/FVC < 70%	TB history (self-reported)	57.2 ± 11.2	NA	57.2 ± 11.2	Low
Wang et al. (46)	China	Cohort study	Men and women aged over 35 years old	3,249	FEV1/FVC < 70%	TB history (self-reported)	52.8 ± 9.4	56.9 ± 9.9	52.7 ± 9.3	Low
Zakaria and Moussa (48)	Egypt	Cross-sectional	Patients previously diagnosed with PTB and had completed anti-tuberculous therapy	50	FEV1/FVC < 70%	TB history—CXR	40.70	NA	NA	Moderate
Chen et al. (17)	China	Cross-sectional	Patients with COPD were included in the study	25	FEV1/FVC < 70%	TB history (self-reported)	NA	NA	NA	High

COPD, Chronic obstructive pulmonary disease; CXR, chest X-ray; FEV1, Forced expiratory volume in one second; FVC, Forced Vital Capacity; GOLD, Global initiative for chronic obstructive lung disease; ICD, International classification of diseases; NA, Not available; PTB, Pulmonary tuberculosis; TB, Tuberculosis.

### Association between history of pulmonary TB and COPD

**Figure 2:**
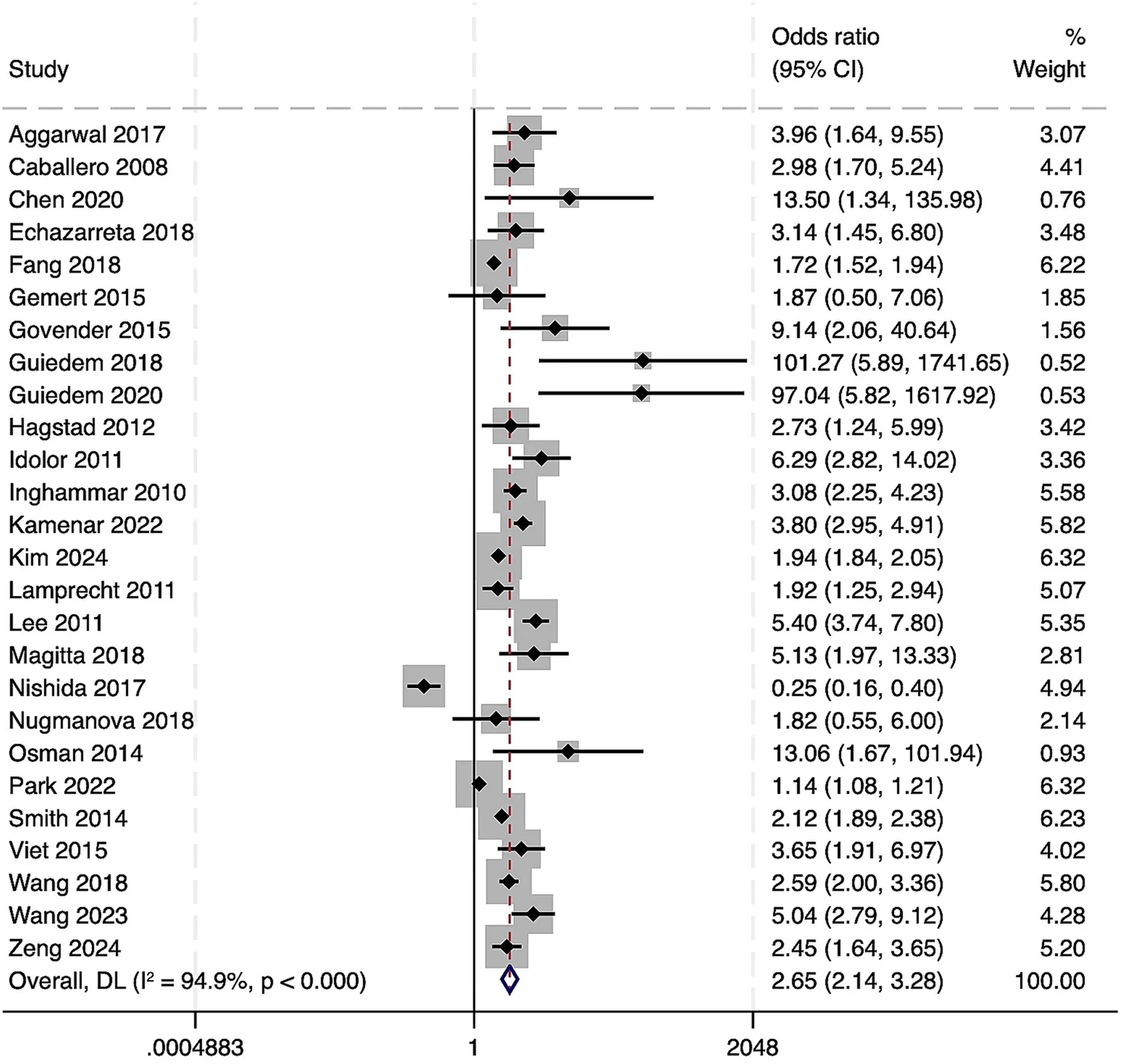
Forest plot showing the association between history of pulmonary TB and COPD. Weights are from random-effects model; continuity correction applied to studies with zero cells.

Across 26 studies (*n* = 1,044,562), prior pulmonary TB was associated with higher odds of COPD: pooled OR = 2.65 (95% CI 2.14–3.28; random-effects). The overall effect was highly significant (*z* = 8.89, *p* < 0.001) (Figure 2). Between-study heterogeneity was substantial (*Q* = 486.69, df = 25, *p* < 0.001; *I*^2^ = 94.9%), with τ^2^ = 0.1889.

**Figure 3:**
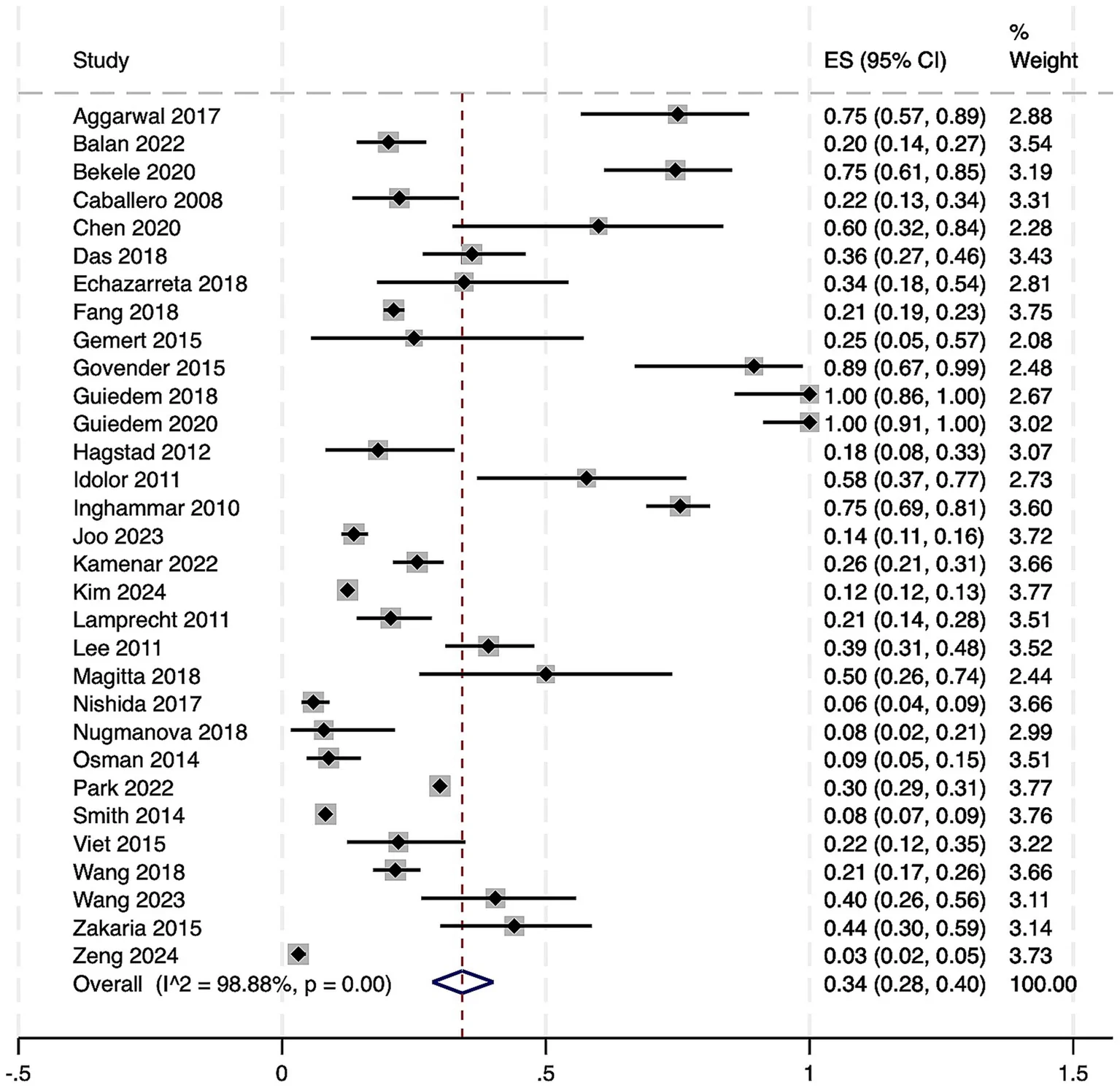
Subgroup analysis based on study design for the association between history of pulmonary TB and COPD.

By study design, pooled random-effects ORs for COPD with prior pulmonary TB were as follows: case–control 4.92 (95% CI 2.30–10.49), cross-sectional 2.64 (1.93–3.61), and cohort 2.46 (1.74–3.48). All subgroup effects were significant (*z* = 4.12, 6.10, and 5.08; all *p* < 0.001). Heterogeneity was negligible in case–control (*I*^2^ = 0.0%; Q = 0.90, *p* = 0.34) but high in cross-sectional (*I*^2^ = 90.9%; Q = 164.35, *p* < 0.001) and cohort studies (*I*^2^ = 97.1%; Q = 242.35, *p* < 0.001). Between-subgroup differences were not significant (Q_between = 2.69, df = 2, *p* = 0.26) (Figure 3).

**Figure 4:**
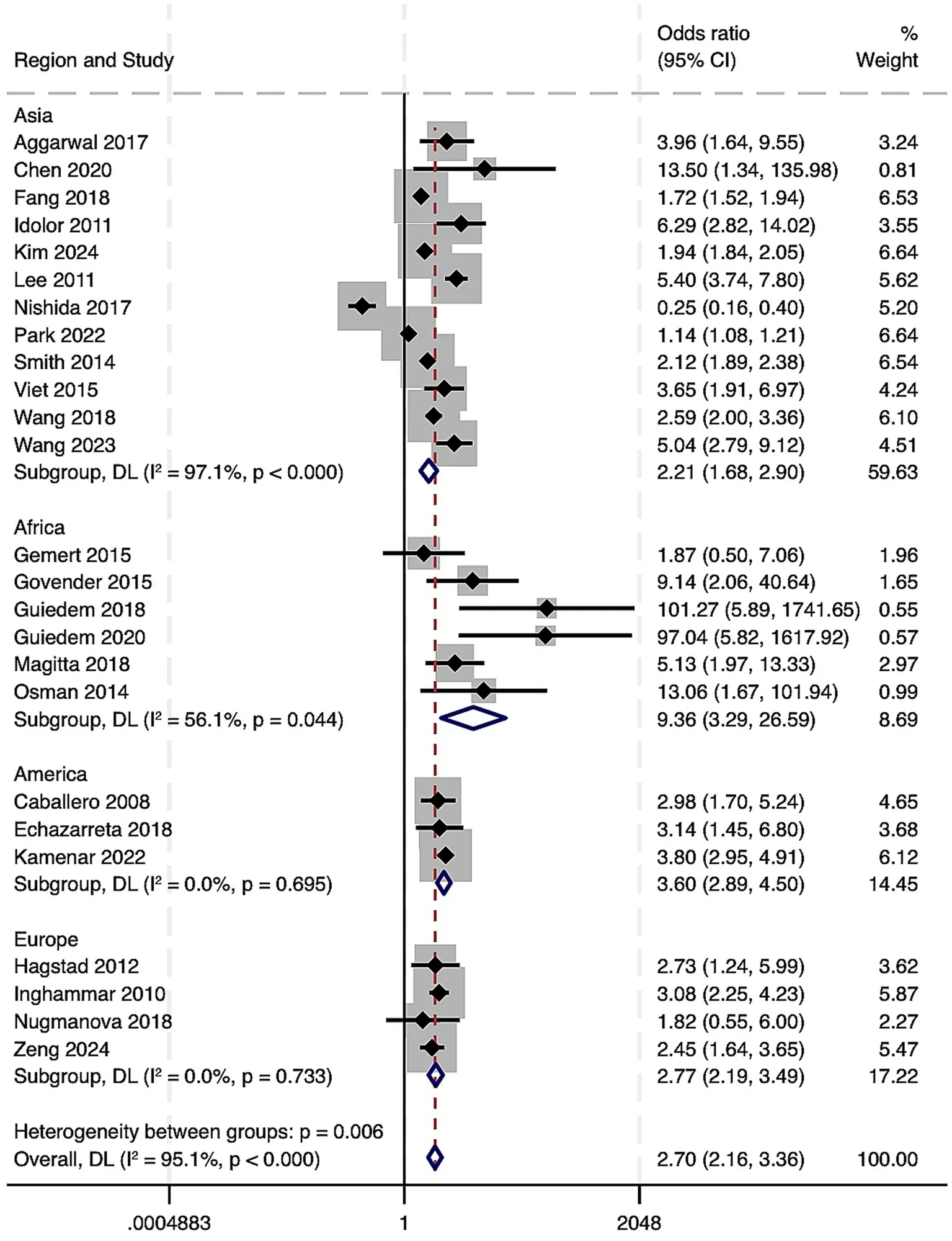
Subgroup analysis based on study region for the association between history of pulmonary TB and COPD. Weights and between-subgroup heterogeneity test are from random-effects model; continuity correction applied to studies with zero cells.

Across 25 studies (*n* = 1,040,271), prior PTB was associated with higher COPD odds in every region. Asia: OR = 2.21 (95% CI 1.68–2.90; *I*^2^ = 97.1%); Africa: OR = 9.36 (3.29–26.59; *I*^2^ = 56.1%). Americas: OR = 3.60 (2.89–4.50; *I*^2^ = 0%); Europe: OR = 2.77 (2.19–3.49; *I*^2^ = 0%); all *p* < 0.001. Between-region heterogeneity was significant (Q_between = 12.50, df = 3, *p* = 0.006) (Figure 4).

Egger’s regression indicated small-study effects and funnel-plot asymmetry (intercept/bias = 2.64, 95% CI 0.60–4.68; *p* = 0.013; slope = 0.36, 95% CI 0.18–0.53; 25 studies). On visual inspection, the funnel plot was asymmetric (Supplementary Figure S1) with greater rightward scatter among smaller, less precise studies, suggesting that smaller studies tended to report larger ORs. This pattern is compatible with publication or selective-reporting bias and/or other small-study effects, though the substantial heterogeneity in the dataset likely contributes to the asymmetry. Leave-one-out sensitivity analysis plot (Supplementary Figure S2) showed that the pooled estimates were robust to the single-study effects with no deviation in magnitude and direction of the pooled estimate.

### Burden of COPD among patients with prior pulmonary TB

**Figure 5:**
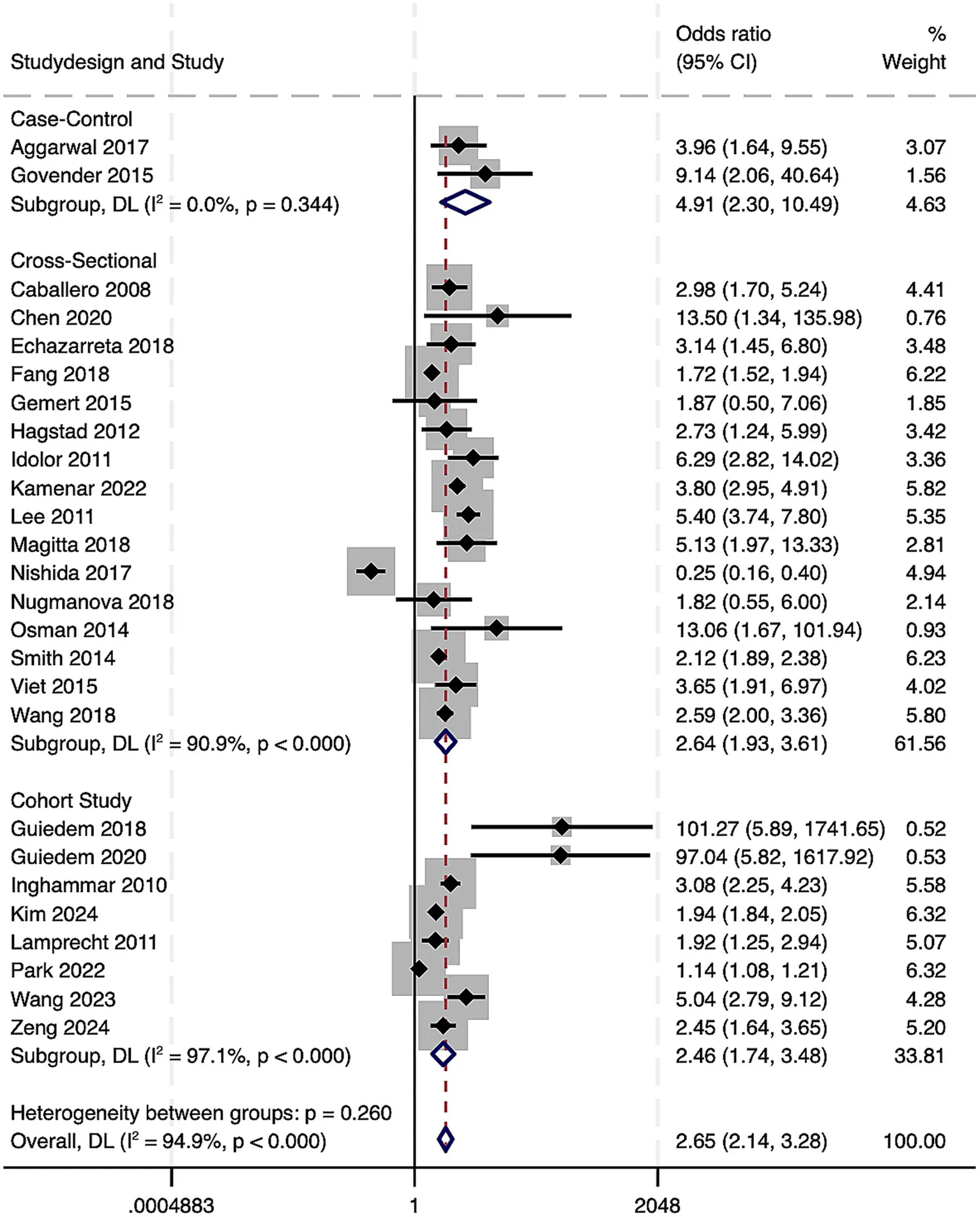
Forest plot showing burden of COPD amongst patients with prior pulmonary TB. Weights and between-subgroup heterogeneity test are from random-effects model; continuity correction applied to studies with zero cells.

Pooled across studies, the prevalence of COPD among people with prior pulmonary TB was 34% (random-effects ES = 0.34; 95% CI 0.28–0.40) (Figure 5). Heterogeneity was extreme (*I*^2^ = 98.9%, *p* < 0.001), with study estimates ranging widely (~3 to 100%), reflecting diverse settings and definitions.

### Burden of prior pulmonary TB among COPD patients

**Figure 6:**
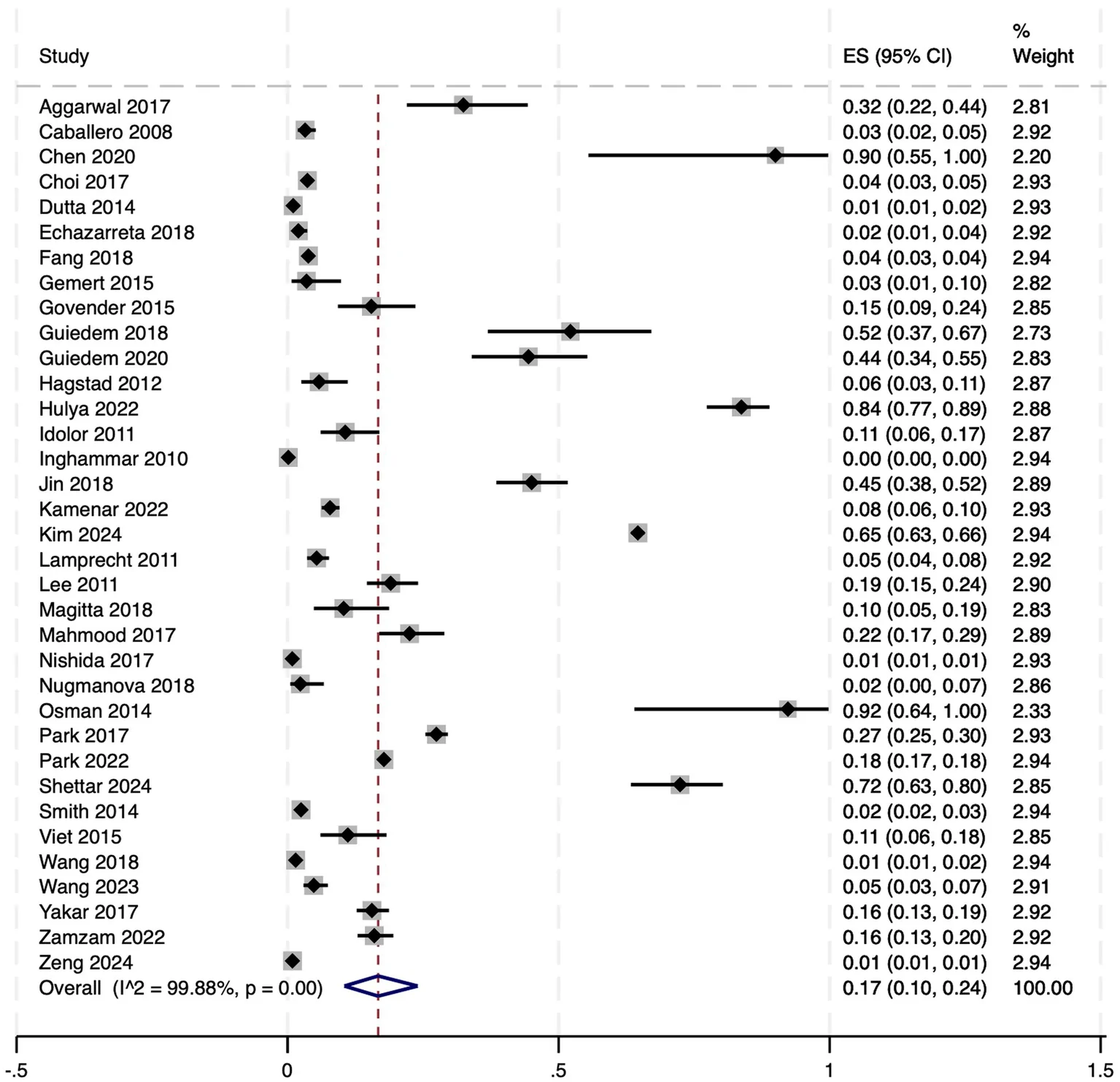
Forest plot showing burden of prior pulmonary TB amongst COPD patients.

Pooled across studies, the prevalence of prior pulmonary TB among people with COPD was 17% (random-effects ES = 0.17; 95% CI 0.10–0.24) (Figure 6). Heterogeneity was very high (*I*^2^ = 99.88%, *p* < 0.001).

## Discussion

This review includes evidence from 40 studies, and data from more than a million participants show that a history of pulmonary TB is significantly associated with COPD. The pooled odds of COPD were two to three times higher among people with prior pulmonary TB, and this association was consistent across different study designs and geographic regions. Case–control studies showed larger effect estimates than cohort and cross-sectional designs. Nonetheless, all three types of designs showed a similar significant association. Across regions, association was found in every region assessed, and it was highest in African studies and persistent in Asia, the Americas, and Europe. Although small-study effects and heterogeneity were found, leave-one-out sensitivity analysis showed that no single study influenced a pooled result, reinforcing that association was robust.

Our findings align with and extend earlier lines of evidence linking tuberculosis survivorship to chronic airflow limitation. Population surveys, such as BOLD, EPI-SCAN, and PLATINO, first drew attention to the excess of spirometric obstruction among individuals reporting previous TB, such as never-smokers and younger adults, suggesting that TB contributes to non-smoking COPD phenotypes (50, 51). Multiple single-country studies from Asia, Africa, and Europe subsequently reproduced this association using administrative codes, spirometry, or clinical diagnosis of COPD (52). Earlier meta-analyses, many of which were dominated by cross-sectional designs and smaller samples, generally reported a two to threefold increase in COPD associated with previous TB (53). This review corroborates that magnitude brings several advances, such as inclusion of large, contemporary cohorts that establish temporality; explicit separation of design-specific effects; and region-specific pooled estimates that illuminate meaningful geographic gradients.

When compared with previous syntheses, our stratified approach shows why effect sizes vary. Case–control studies, which often include samples from clinics and may rely on retrospectively reported pulmonary TB, tend to produce larger estimates, likely reflecting the combination of biological and design factors, such as differential recall, spectrum bias, and/or differential referral of severe cases. Cross-sectional surveys frequently show heterogeneous effects because they combine diverse COPD definitions (fixed ratio vs. lower limit of normal; pre vs. post-bronchodilator spirometry) and rely variably on self-reported pulmonary TB. Cohort studies mitigate such reverse causation and clarify that temporality and pooled effects are moderately lower but remain significant, indicating causal interpretation. Pattern, which is large, but variable cross-sectional effects and significant, directionally consistent cohort effects mirrors the trajectory in broader literature as methodological quality has improved. Earlier work often treated prior TB as a covariate rather than a single exposure; by foregrounding it, our synthesis highlights the epidemiologic salience of TB survivorship within COPD case-mix, which has implications for phenotype recognition and treatment tailoring (54, 55).

Importantly, the accumulated evidence base now includes several large registry-based cohorts and biobank analyses showing elevated incident COPD risk after TB, even after adjustment for smoking and other confounders (56, 57). These higher-quality designs counterbalance selective reporting by smaller studies and reinforce that the association is not spurious. The robustness of our pooled estimate to leave-one-out diagnostics further mitigates concerns that the signal is driven by a handful of outliers.

Finally, our results complement and extend the emerging concept of “TB-associated COPD” as a clinically meaningful phenotype. Prior literature has emphasized that post-TB airflow obstruction often coexists with radiographic bronchiectasis, irregular emphysema, and small-airway dysfunction, features that differ from classic, smoke-related centrilobular emphysema (5, 58, 59). The strength and consistency of the association in the present synthesis, coupled with sizeable burdens in both directions of the exposure–outcome relationship, support the view that TB survivorship is a major, distinct pathway to COPD.

Several complementary biological and social mechanisms plausibly account for the strong association between a history of pulmonary tuberculosis and subsequent COPD. Active TB incites intense, protease-rich inflammation within the lung parenchyma and small airways (60). Neutrophil elastase, matrix metalloproteinases, and dysregulated host reparative responses erode alveolar attachments, narrow small airways, and distort the bronchial tree. Even when microbiologic cure is achieved, healing commonly proceeds through fibrosis and traction bronchiectasis; these changes fix airflow limitation and perpetuate mucus stasis and recurrent infection (61, 62). Cavitation and bronchiectatic segments can produce regional hyperinflation and air-trapping that are physiologically indistinguishable from classic obstructive disease, but with a distribution and radiologic appearance that differ from smoking-predominant centrilobular emphysema.

However, important limitations still exist in this review. Heterogeneity was high for primary association, indicating significant variation beyond chance. A large amount of this heterogeneity came from inconsistent definitions of both exposure and outcome. Several studies relied on self-reported TB or chest X-ray descriptors that may misclassify healed TB, while COPD ascertainment ranged from administrative codes to pre and post-bronchodilator spirometry using fixed-ratio or lower-limit-of-normal thresholds. Small-study effects and funnel-plot asymmetry suggest that selective reporting or publication bias may inflate the magnitude of association in parts of the literature.

The results have important implications for clinicians and TB programs in the implementation of their post-treatment care. First, a history of pulmonary TB should be treated as a major COPD risk factor and documented routinely in respiratory assessment, mainly in high-burden countries. Incorporating TB history into risk stratification and case-finding algorithms can improve yield amongst adults presenting with symptoms like chronic cough or dyspnoea. Second, spirometry, which is ideally done after bronchodilator and interpreted with age-appropriate reference equations, should be available to all TB survivors shortly after their treatment completion, with a referral pathway to respiratory care for those with airflow obstruction and/or persistent symptoms. Where capacity of spirometry is limited, pragmatic symptom screening combined with peak expiratory flow or simple oscillometer can support triage.

## Conclusion

This review shows that there is a significant association between a previous history of pulmonary TB and COPD irrespective of study designs and geographical regions. The consistency of the direction of association, robustness of the pooled estimate in influence analyses, and alignment with pathophysiologic understanding support the interpretation that TB survivors are important contributors to the global burden of COPD. Although heterogeneity is high and small-study effects are present, the association still persists in large cohorts that provide evidence supporting causality, and burden estimates underscore the real-world consequences where a significant number of TB survivors live with COPD, and a minor proportion of people with COPD have a history of TB.

## Data Availability

The data analyzed in this study is subject to the following licenses/restrictions: data will be shared on request to author. Requests to access these datasets should be directed to lyy250726@sina.com.

## Supplementary material

SUPPLEMENTARY FIGURE S1 Funnel plot for association between history of pulmonary TB and COPD.

SUPPLEMENTARY FIGURE S2 Leave one out sensitivity analysis plot for association between history of pulmonary TB and COPD.
